# Geometric compensation applied to image analysis of cell populations with morphological variability: a new role for a classical concept

**DOI:** 10.1038/s41598-018-28570-z

**Published:** 2018-07-06

**Authors:** Joana Figueiredo, Isabel Rodrigues, João Ribeiro, Maria Sofia Fernandes, Soraia Melo, Bárbara Sousa, Joana Paredes, Raquel Seruca, João M. Sanches

**Affiliations:** 1 Instituto de Investigação e Inovação em Saúde (i3S), Porto, Portugal; 2 Institute of Molecular Pathology and Immunology of the University of Porto (IPATIMUP), Porto, Portugal; 3 Institute for Systems and Robotics (ISR/IST), LARSyS, Bioengineering Department, Instituto Superior Técnico, Universidade de Lisboa, Lisboa, Portugal; 4 Medical Faculty of the University of Porto, Porto, Portugal

## Abstract

Immunofluorescence is the gold standard technique to determine the level and spatial distribution of fluorescent-tagged molecules. However, quantitative analysis of fluorescence microscopy images faces crucial challenges such as morphologic variability within cells. In this work, we developed an analytical strategy to deal with cell shape and size variability that is based on an elastic geometric alignment algorithm. Firstly, synthetic images mimicking cell populations with morphological variability were used to test and optimize the algorithm, under controlled conditions. We have computed expression profiles specifically assessing cell-cell interactions (IN profiles) and profiles focusing on the distribution of a marker throughout the intracellular space of single cells (RD profiles). To experimentally validate our analytical pipeline, we have used real images of cell cultures stained for E-cadherin, tubulin and a mitochondria dye, selected as prototypes of membrane, cytoplasmic and organelle-specific markers. The results demonstrated that our algorithm is able to generate a detailed quantitative report and a faithful representation of a large panel of molecules, distributed in distinct cellular compartments, independently of cell’s morphological features. This is a simple end-user method that can be widely explored in research and diagnostic labs to unravel protein regulation mechanisms or identify protein expression patterns associated with disease.

## Introduction

Immunofluorescence (IF) microscopy is a widely used technique that uses fluorescent-labelled markers to visualize the distribution of proteins, glycoproteins and other molecular targets in intracellular structures, at the cellular level or at the tissue level^1,2^. In the last years, different approaches have been developed to extract quantitative features from IF images and, in this way, to better understand the most complex cellular mechanisms^3,4^. Image acquisition modalities, such as time-lapse microscopy, confocal laser scanning microscopy (CLSM) and spinning disk microscopy can offer quantitative analysis of a target protein; nonetheless, those techniques rely on measurements of total fluorochrome intensity, regardless of its distribution in an image or in a selected region^5–7^.

Recently, we have developed a bioimaging tool to assess the patterns of expression of *CDH1* germline missense variants associated to a cancer syndrome^8^. In that approach, the major analytical challenge was related to the heterogeneous morphology of cells in IF images. In fact, within the same cell population, it is possible to identify cells with very different shapes and sizes due to DNA replication error/mutations, epigenetic alterations, independent clonal evolution or different cell cycle stages^9,10^.

Different morphological features will give rise to a high variability in the expression profiles, impairing the extraction of a representative overview/map of a particular target within a heterogeneous cell population. To overcome this constraint on endogenous cell-to-cell differences, we developed a geometric compensation model specific for *in situ* IF applications. Geometric compensation is a common procedure in several image analysis modalities, and typically consists on the estimation of rigid or non-rigid transformations to make the objects under alignment as similar as possible in terms of shape and size^11–14^. This technique is essential for image reconstruction and fusion by improving the resolution of the raw data and increasing image analysis accuracy^11–14^.

In this report, we describe an analytical pipeline which includes the extraction of internuclear (IN) and radial (RD) fluorescence profiles, and their accurate alignment. Specifically, the method applies a Bayesian non-rigid alignment algorithm and an automatic outlier rejection strategy to a large number of individual profiles, minimizing the undesired effect of size and shape variability within the cell population. In this way, the algorithm generates a final profile which is a distorted version of an ideal unknown profile, representative of all cells analysed within an IF image.

To experimentally validate our strategy, we applied this new algorithm to IF images of real cell cultures stained with E-cadherin, a key cell-cell adhesion molecule; tubulin, a major component of the eukaryotic cytoskeleton; and Mitotracker that labels mitochondria, which are complex cytoplasmic organelles responsible for the generation of energy in cells^15–20^. Altogether, prototype markers of membrane, cytoplasmic and organelle-specific moieties, representative of distinct cellular compartments, were incorporated in our validation.

## Results

### Extraction of synthetic expression profiles

In imaging analysis, cellular morphological heterogeneity is a major challenge that needs to be addressed to obtain an accurate quantitative map of a tagged molecule in a cell population. Herein, we took advantage of an alignment algorithm to minimize the variability of synthetic and real fluorescence profiles, demonstrating the accuracy of our approach to achieve a typical picture and a precise expression profile of proteins in the populations analysed.

**Figure 1:**
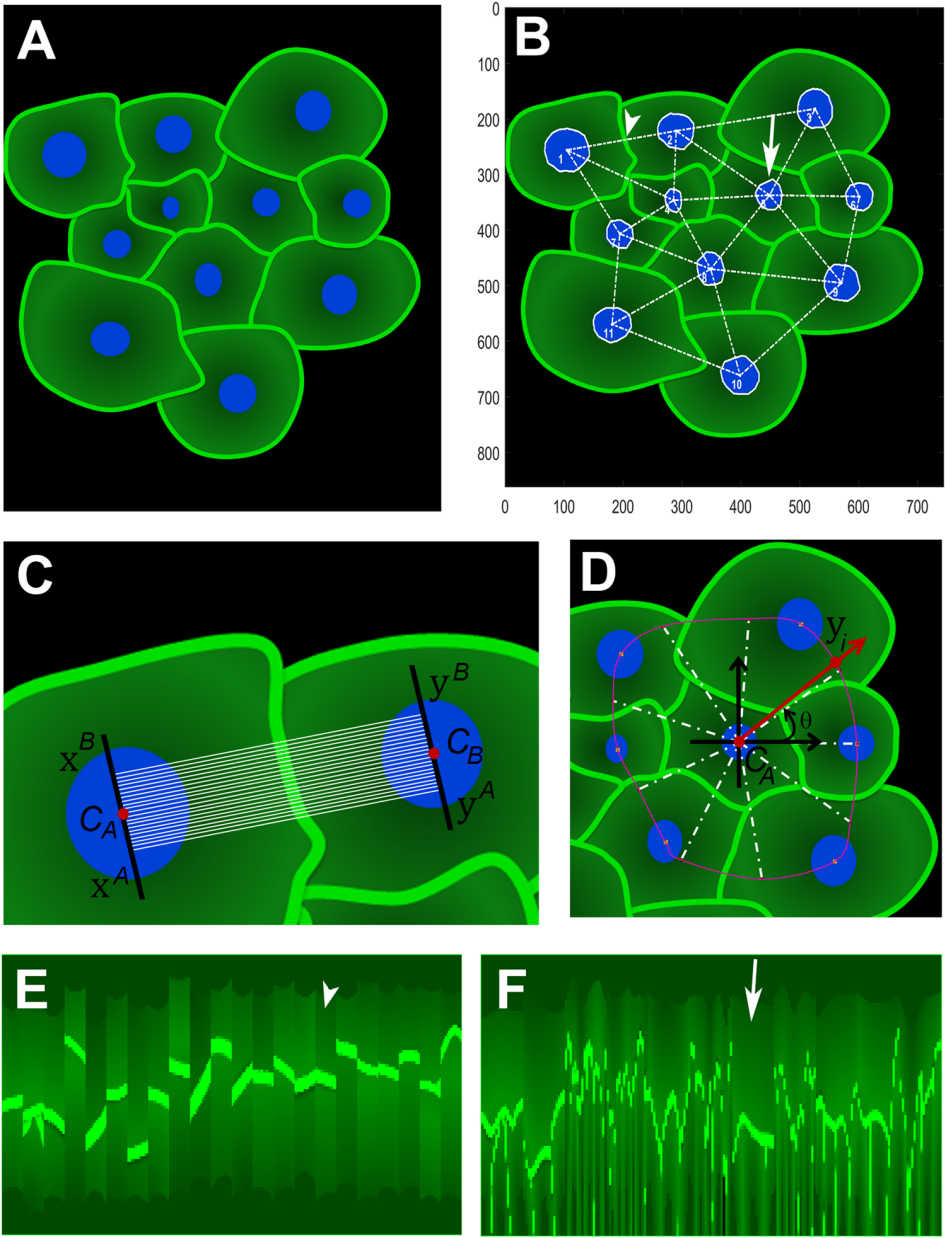
Profiling internuclear (IN) and radial (RD) expression in heterogeneous cell populations. (**A**) Synthetic image mimicking cellular heterogeneity, concerning size and morphology. (**B**) Automatic selection and networking of cells through nuclei segmentation and calculation of their geometric centroids. Arrowhead represents IN profiles and arrow corresponds to RD analysis. (**C**) IN profiles capture signal intensities occurring between two contiguous cells. (**D**) RD profiles encompass fluorescence patterns that spread from a nucleus centroid and cover the total area of a single cell. (**E**,**F**) IN and RD intensity maps, respectively, obtained from the original synthetic image presented in (**A**).

To achieve our goal, the strategy applied involved a number of specific steps. First, cells composing synthetic images mimicking heterogeneous cultures/tissues were automatically selected and connected using a bioimaging tool previously developed by our group (Fig. 1A,B)^21^. The algorithm generates a nucleus-nucleus network representing cell distribution across the image, in which the nodes are the geometric centres of the cell nuclei and the edges represent the neighbouring relation between them. The Delaunay tessellation algorithm automatically groups neighbour nodes in three element clusters (triangles), minimizing their total area and eccentricity. The networks are independent of the non-regular distribution of cells and avoid the need of repetitive and predictive patterns to define a neighbouring system. Noteworthy, for an accurate intensity mapping, cells should be confluent in a way that only neighbouring/adjacent cells are associated in triplets, forming a contiguous diagram. The presence of empty space between cells could lead to an erroneous interpretation of the data.

The connections retrieved from this networking process were then used to extract IN and RD profiles from cell pairs and individual cells, respectively. As showed in Fig. 1C, IN profiles consist of a set of quasi-parallel segments around the main axis linking the geometrical centres of two neighbour nuclei and measure fluorescence intensities occurring between two contiguous cells. This output is of particular relevance to evaluate proteins located at the plasma membrane or in specific cellular organelles.

In order to extract IN profiles, we have considered the diameters of the nuclei perpendicular to the IN axis, linking both geometric centres of the nuclei and their interceptions with the nuclei, x^A^, x^B^, y^A^ and y^B^, where ${\rm{x}},\,{\rm{y}}\in {{\mathbb{R}}}^{2}$. The starting and ending points, x_i_ and y_i_, of the i^th^ straight line/profile within the beam are the following:

1 $$\{\begin{array}{c}{{\rm{x}}}_{{\rm{i}}}={{\rm{x}}}^{{\rm{A}}}+\frac{{\rm{i}}}{{\rm{n}}-1}({{\rm{x}}}^{{\rm{B}}}-{{\rm{x}}}^{{\rm{A}}})\\ {{\rm{y}}}_{{\rm{i}}}={{\rm{y}}}^{{\rm{A}}}+\frac{{\rm{i}}}{{\rm{n}}-1}({{\rm{y}}}^{{\rm{B}}}-{{\rm{y}}}^{{\rm{A}}})\end{array}$$

where ${\rm{i}}=0\,\cdots \,{\rm{n}}-1$ and n, the number of profiles within the beam, is typically n = 10.

The intensity of the j^th^ pixel of the i^th^ profile is G(p_i_(j)), where ${\rm{G}}(\,.\,)$ is the image intensity of the channel of interest at the location

2 $${{\bf{p}}}_{{\rm{i}}}({\rm{j}})={{\bf{x}}}_{{\rm{i}}}+\frac{{\rm{j}}}{{{\rm{m}}}_{{\rm{i}}}-1}({{\bf{y}}}_{{\rm{i}}}-{{\bf{x}}}_{{\rm{i}}})$$

Here, ${\rm{j}}=0\,\cdots \,{{\rm{m}}}_{{\rm{i}}}-1$. m_i_ is the length of the i^th^ profile, which is typically the distance between the geometric centres of the nuclei, c_A_ and c_B_, ${{\rm{m}}}_{{\rm{i}}}=\Vert {{\rm{c}}}_{{\rm{A}}}-{{\rm{c}}}_{{\rm{B}}}\Vert$. Since the IN distances were different from pair to pair of cells and the next step was to package them in a single N × M matrix, where N is the total number of profiles and M is the length of them, an interpolation procedure with bi-cubic functions was performed over each extracted profile to normalize their length to M samples.

In contrast, RD profiles were designed to capture the intensity pattern throughout the cytoplasm of a single cell (Fig. 1D) and could be very useful for the analysis of cytoplasmic proteins. Indeed, the RD profiles correspond to a set of m equi-spaced angular profiles anchored at the centres of the nuclei. The length of each profile within the set depends on the neighbouring configuration in the vicinity of the cell. As shown in Fig. 1D, an interpolation spline passing by the adjacent cells defines the limit and the length of each profile. Further, as described for IN profiles, the different lengths of RD profiles were normalized using a bi-cubic interpolation operation, allowing the packaging of all profiles (from all cells) in a N × M matrix. Here, M is the normalized length of the profiles, $N={N}_{c}m$ is the total number of RD profiles and *N*_*c*_ is the number of cells in the image.

The intensity of the j^th^ pixel from the i^th^ profile, extracted from the k^th^ cell, is G(p_i_(j)) where ${\rm{G}}(\,.\,)$ is the image intensity at the location

3 $${{\bf{p}}}_{{\rm{i}}}({\rm{j}})={{\bf{c}}}_{k}+\frac{{\rm{j}}}{{{\rm{r}}}_{{\rm{i}}}-1}({{\bf{y}}}_{{\rm{i}}}-{{\bf{c}}}_{k})$$

Here, ${\rm{j}}=0\,\cdots \,{{\rm{r}}}_{{\rm{i}}}-1.\,{{\rm{r}}}_{{\rm{i}}}$ is the length of the i^th^ profile, which corresponds to the distance from the centre of the cell to the interception of the corresponding radius vector (Fig. 1D) with the spline that passes through the cell centroids.

As demonstrated in Fig. 1E,F, IN and RD original maps were successfully obtained from synthetic IF images following this pipeline. However, their huge irregularity prevents obtaining a clear picture of the expression pattern represented in the original image.

### Alignment of synthetic profiles and image reconstruction

IN and RD profile maps obtained in the previous step are composed by stacks of profiles extracted from different cells and pairs of cells, with different sizes and shapes. Therefore, the panel of profiles is highly heterogeneous and, as expected, it is not possible to directly extract their typical protein distribution.

**Figure 2:**
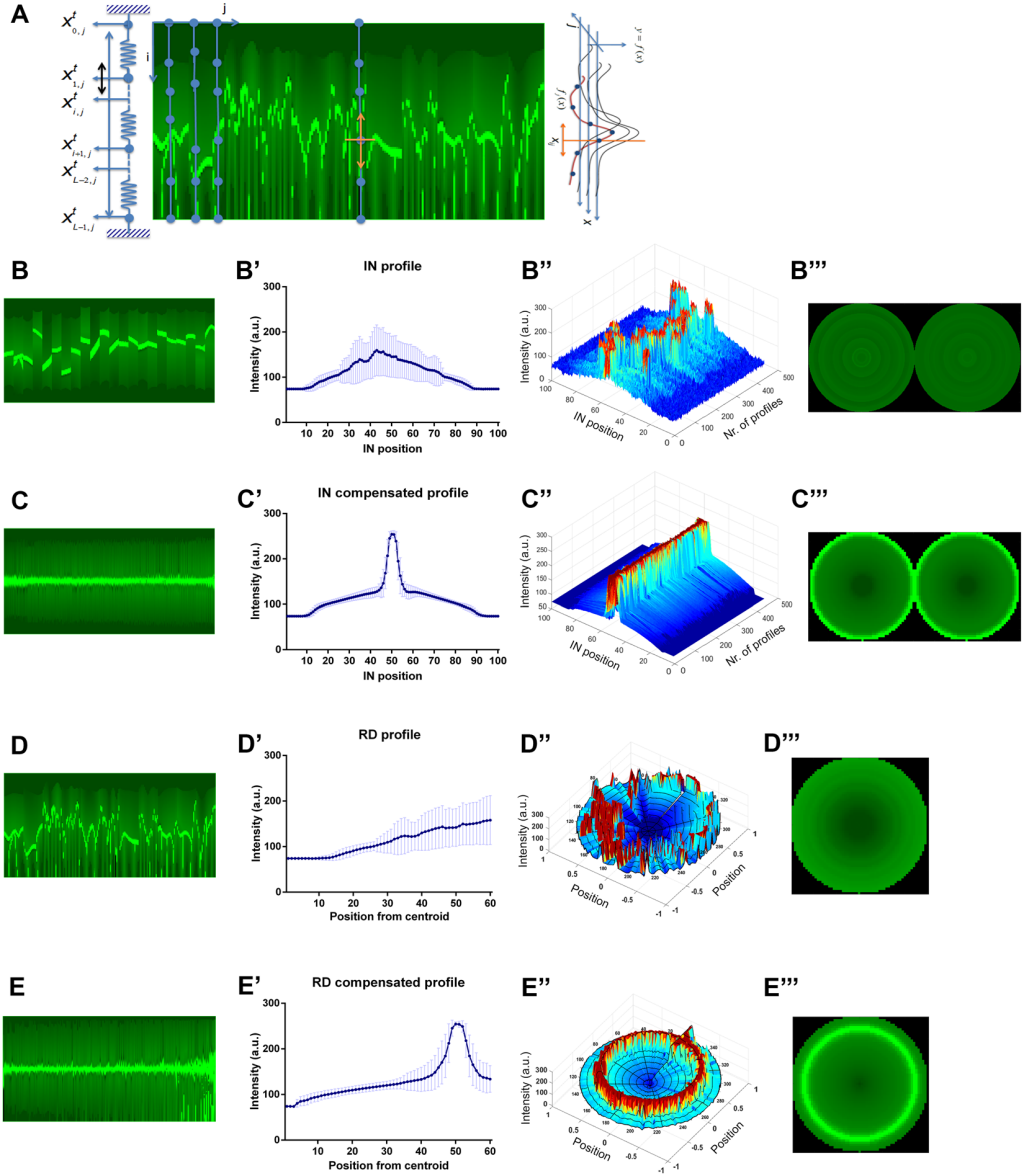
Geometrically compensated profiles reproduce signal patterning of synthetic images. (**A**) Strategy for geometric alignment. An iteration model of intensity’s adjustment was applied to each profile from the intensity map, imposing a tension regularization term and, simultaneously, a similarity driven force. (**B**,**C**) non-compensated and compensated IN maps, respectively, extracted from the same synthetic image. (B’,C’) Mean and standard deviation (SD) of non-aligned and aligned IN profiles. (B”) and (C”) 3D overviews of all extracted IN profiles. (B”’,C”’) Virtual cell pairs illustrating signal distribution in non-aligned and aligned IN profiles. A similar analysis is presented in panels D and E for the corresponding non-compensated and compensated RD profiles. a.u., arbitrary units.

To overcome the variability of IN and RD maps, a geometric compensation method was developed (Fig. 2A), assuming that all profiles within each map are distorted versions of an unknown ideal profile that is representative of the protein distribution in the whole cell population.

For that purpose, we established an *N* × *M* matrix with $Y=\{{y}_{i,j}\}$ representing the map of profiles and $X=\{{x}_{i,j}\}$ holding the same dimensions of *Y*. The matrix contains the normalized locations of the intensities, *y*_*i,j*_, along the profiles, where ${x}_{i,j}\in [0,1]$. Importantly, the distance between cells is not constant, so the length of the extracted profiles may be different, especially in the case of IN profiles. Therefore, profiles were interpolated, using a bi-cubic interpolation function, and converted into *N* length vectors suitable to be packed side-by-side in the matrix *Y*.

Matrix *X* contains the initial locations of the observations, *x*_*i,j*_, that we have assumed to be evenly distributed in the interval [0, 1] according to ${x}_{i,j}=i/(N-1)$ with $i=0,\ldots ,N-1$. Each *N* length column of *Y*, **y**_*j*_, was also assumed to be a distorted and non-uniformly sampled version of an ideal continuous profile, representative of the entire population, $f(x):{\rm{\Omega }}\to R$, where ${\rm{\Omega }}=[0,1]$.

The distortion of each profile was described by the unknown monotonic function *g*_*j*_(*x*). The algorithm is based on the adjustment of the initial locations of the observations, *x*_*i,j*_, in order to estimate de inverse of *g*_*j*_(*x*) and, consequently, the real locations of the observations, ${x}_{i,j}^{\ast }={g}_{j}^{-1}({x}_{i,j})$.

The ideal profile is, thus, a finite dimension continuous function, *f*(*x*, ***c***), where ${\boldsymbol{c}}=\{{c}_{k}\}$ is a vector of coefficients to be calculated. The estimation of **c**, as well as the compensated locations, ${x}_{i,j}^{\ast }={g}_{j}^{-1}({x}_{i,j})$, were formulated according the following optimization problem:

4 $${[{\bf{c}},X]}^{\ast }=arg\,{{\rm{\min }}}_{{\boldsymbol{c}},X}\,E(X,{\bf{c}},Y)$$

Here the energy function to be minimized is composed by three terms:

5 $$E(X,{\bf{c}},Y)={E}_{Y}(X,{\bf{c}},Y)+{E}_{c}({\bf{c}})+{E}_{X}(X)$$

the data fidelity term *E*_*Y*_; the regularization term for **c**, *E*_*c*_; and the prior term for the observed locations, *E*_*X*_. This equation will induce similarity between neighbouring profiles within the map and, consequently, the alignment and geometric compensation of the profiles. A number of assumptions need to be highlighted and include:

#### Ideal Profile

The unknown function that describes the ideal profile to be estimated is assumed to be a finite dimension continuous function described by a linear combination of *L* ideal interpolation functions, ${\varphi }_{k}(x)=sinc(x/{\Delta }-k)$ with ${\Delta }={(L-1)}^{-1}$ and $k=0,\,1,\,\ldots ,\,L\,-\,.$

6 $$f(x)=\sum _{k=0}^{L-1}{c}_{k}{\varphi }_{k}(x)={\varphi }^{T}(x){\bf{c}}$$

Here, $\varphi (x)={\{{\varphi }_{0}(x),{\varphi }_{1}(x),\ldots ,{\varphi }_{L-1}(x)\}}^{T}$ is an *L* length column vector containing the interpolation functions computed at location *x*, and ${\bf{c}}={\{{c}_{0},{c}_{1},\ldots ,{c}_{L-1}\}}^{T}$ is an unknown *L* length column vector of coefficients that needs to be estimated.

Because the IN profiles describe the typical intensity distributed from the cell A to the cell B, which is the same from B to A, the vector **c** should be symmetric by imposing ${\bf{c}}=P\tilde{c}$, where $\tilde{c}$ is a (*L*/2) length vector and *P* is the following *L* × (*L*/2) matrix

7 $$P=[\begin{array}{ccccc}1 & 0 & 0 & \ldots  & 0\\ 0 & 1 & 0 & \ldots  & 0\\ \vdots  & \vdots  & \vdots  & \ldots  & \vdots \\ 0 & 0 & \ldots  & 1 & 0\\ 0 & 0 & \ldots  & 0 & 1\\ 0 & 0 & \ldots  & 1 & 0\\ \vdots  & \vdots  & \vdots  & \ldots  & \vdots \\ 1 & 0 & 0 & \ldots  & 0\end{array}]$$

The ideal profile can be described as following

8 $$f(x)={\varphi }^{T}(x)P\tilde{c}$$

Noteworthy, the IN ideal profile undergo symmetry constraints, using *P* as defined in (7), while the RD ideal profile is not subjected to symmetry constraint, *P* = *I*_*L*_, where *I*_*L*_ is the L × L identity matrix.

#### Data fidelity term

The common used additive white Gaussian noise (AWGN) model^22^ leads to the following data fidelity term

9 $${E}_{Y}(X,{\bf{c}},Y)=\sum _{i,j}{\omega }_{i,j}(f{({x}_{i,j}-{y}_{i,j})}^{2})$$

where *ω*_*i,j*_ are outlier indicators,

10 $$\omega =\{\begin{array}{ll}1 & {\rm{valid}}\,{\rm{observation}}\\ 0 & {\rm{outlier}}\end{array}$$

The indicators are adaptively computed along the iterative process of estimation. In case the distance of *j*^*th*^ profile to the current estimation of *f*(*x*), $\,||f({x}_{j})-{y}_{j}{||}_{2}^{2}$, is larger than a given threshold, the indicators corresponding to that column are set to zero, *ω*_*i,j*_ = 0 with $0\le i\le N-1$. Hence, the profile is classified as an outlier and is not used to estimate *f*(*x*). However, its location, x_*j*_, is still updated and, in future iterations, it can be re-classified as valid data and be included in the estimation of *f*(*x*).

From the models commonly used to describe intensities in fluorescence images, the Poisson distribution model was preferred, as the data is obtained with photon-limited and counting-based image acquisition processes, where a small amount of detected radiation and a huge optical/electronics amplification is involved^23^. Further, assuming the independence between observations, the data fidelity term is symmetric of the log-likelihood function

11 $$\begin{array}{rcl}{E}_{Y}({\rm{x}},{\bf{c}},Y) & = & -\,\mathrm{log}\,P(y|\,f(x,{\bf{c}}))\\  & = & -\sum _{i,j}\,\mathrm{log}\,p({y}_{i,j}|\,f({y}_{i,j},{\bf{c}}))\end{array}$$

where $p(y|f(x,{\bf{c}}))$ is the Poisson distribution $p(y|f(x))=(f{(x)}^{y}/y!){e}^{-f(x)}$, with parameter *f*(*x*, **c**), resulting in the following data fidelity term:

12 $${E}_{Y}({{\rm{x}}}_{j},{\bf{c}},Y)=\sum _{i,j}{\omega }_{j}[f({x}_{i,j})-{y}_{i,j}\,\mathrm{log}(f({x}_{i,j}))]$$

#### Function regularization

The solution of (4) that defines the function *f*(*x*, **c**), representing the ideal population profile, was regularized using a quadratic penalty term to force smoothness of *f*(*x*) defined in (6),

13 $${E}_{c}({\bf{c}})=\alpha \sum _{k=0}^{L-1}{({c}_{k}-{c}_{k-1})}^{2}=\alpha {{\bf{c}}}^{T}{{\rm{\psi }}}_{L}{\bf{c}}$$

Here,

14 $${{\rm{\psi }}}_{L}={\theta }_{L}^{T}{\theta }_{L}$$

with the following *L* × *L* difference operator

15 $$\theta =[\begin{array}{ccccc}1 & -1 & 0 & \ldots  & 0\\ -1 & 1 & 0 & \ldots  & 0\\ 0 & -1 & 1 & \ldots  & 0\\ \vdots  & \vdots  & \vdots  & \vdots  & \vdots \\ 0 & 0 & \ldots  & -1 & 1\end{array}]$$

Similarly, the smoothing regularization prior term for **b** is

16 $${E}_{b}({\bf{b}})=\alpha \sum _{k=0}^{L/2-1}{({b}_{k}-{b}_{k-1})}^{2}=\alpha {{\bf{b}}}^{T}{{\rm{\psi }}}_{L/2}{\bf{b}}$$

#### Locations regularization

The optimization of the data fidelity term (11) concerning the observation locations, *x*_*i,j*_, is an *ill-posed* problem that also needs to be regularized. A trivial solution would be the collapsing of all locations at the same point. So, to avoid that, the limits were kept fixed (not updated) – *x*_0,*j*_ = 0 and *x*_*N*-1,*j*_ = 1 – and a regularization term was introduced by imposing a tension force between the neighbouring locations in each profile

17 $$\begin{array}{rcl}{E}_{X}(X) & = & \beta \sum _{i,j}{({x}_{i,j}-{x}_{i-1,j})}^{2}\\  & = & \beta Tr[{X}^{T}{{\rm{\psi }}}_{N}X]\end{array}$$

Here, *Tr* denotes the *Trace* operator and ψ_*N*_ is the *N* × *N*, as defined in (14) and demonstrated in Fig. 2A. At the end, the overall energy to be minimized is the following

18 $$E(X,{\bf{c}},Y)={E}_{Y}(X,{\bf{c}},Y)+{\rm{\alpha }}{{\bf{c}}}^{T}{{\rm{\psi }}}_{L}{\bf{c}}+\beta Tr[{X}^{T}{{\rm{\psi }}}_{N}X]$$

Optimization: The vectors of coefficients, **c**, as well as the location of the observations ${\bf{X}}=\{{x}_{i,j}\}$ were estimated along an iterative process. Further, the steps for the minimization of a global energy function *E*(*X*, **c**, *Y*), alternate regarding **c** and *X* until a stopping criterion is met,

19 $${{\bf{c}}}^{t+1}={\rm{\arg }}\mathop{\min }\limits_{{\bf{c}}}E({X}^{t},{\bf{c}},Y)$$

20 $${X}^{t+1}={\rm{\arg }}\,{{\rm{\min }}}_{X}\,E(X,{{\bf{c}}}^{t+1},Y)$$

The minimization step (19) is performed by solving ${\nabla }_{{\bf{c}}}E({X}^{t},{\bf{c}},Y)=0$. For gradient computation purposes, the data fidelity terms (9) and (12) can be defined as follows

21 $${E}_{Y}(X,{\bf{c}},Y)={({{\rm{\varphi }}}^{T}({\rm{x}}){\bf{c}}-y)}^{T}\sum ({{\varphi }}^{T}({\rm{x}}){\bf{c}}-y)$$

Here, ${\rm{x}}=vect(X)=\{{x}_{k}\}$ is the vectorization of matrix *X*, ϕ(x) is an *L* × *NM* matrix where each column contains the vectors *ϕ*(*x*_*k*_), and $\sum \,=\,\{{\sigma }_{i,j}\}=\{{\omega }_{i,j}{\gamma }_{i,j}\}$ is an *NM* × *NM* diagonal matrix with

22 $${\gamma }_{i,j}=\{\begin{array}{cc}1 & AWGN\\ 1/(2f({x}_{i,j})+{\epsilon }) & {\rm{Poisson}}\end{array}$$

*ω*_*i,j*_ are the outlier indicators (as defined above) and ${\epsilon }={10}^{-6}$ is a small constant to prevent division by zero. The minimization step (19) is then performed:

23 $$\begin{array}{rcl}{\nabla }_{{\bf{c}}}E(X,{\bf{c}},Y) & = & {\nabla }_{{\bf{c}}}{E}_{Y}(X,{\bf{c}},Y)+{\nabla }_{{\bf{c}}}{E}_{{\bf{c}}}({\bf{c}})\\  & = & ({\rm{\varphi }}({\rm{x}}){\rm{\Sigma }}\,{{\rm{\varphi }}}^{T}({\rm{x}})+\alpha {{\rm{\psi }}}_{L}^{T}){\bf{c}}-\varphi ({\rm{x}}){\rm{\Sigma }}y=0\end{array}$$

This allows the generation of the following recursion

24 $${{\bf{c}}}^{t+1}={({\rm{\varphi }}({{\rm{x}}}^{t})\sum {{\rm{\varphi }}}^{T}({{\rm{x}}}^{t})+\alpha {{\rm{\psi }}}_{L}^{T})}^{-1}\varphi ({{\rm{x}}}^{t})\sum y$$

where x^*t*^ is the current estimate of x.

By including the symmetry constraint (8) used in the IN profiles, the following coefficients can be obtained

25 $${\tilde{{\boldsymbol{c}}}}^{t+1}={({P}^{T}{\rm{\varphi }}({{\rm{x}}}^{t})\sum {{\rm{\varphi }}}^{T}({{\rm{x}}}^{t})P+\alpha {{\rm{\psi }}}_{L/2})}^{-1}{P}^{T}\varphi ({{\rm{x}}}^{t})\sum y$$

Subsequently, the minimization step (20), where the observation locations are updated, is applied by solving the following equation:

26 $$\frac{\partial E}{\partial {x}_{i,j}}={z}_{i,j}+\beta ({x}_{i,j}-{\bar{x}}_{i,j})=0$$

Here, ${z}_{i,j}={\gamma }_{i,j}[f({x}_{i,j})-{y}_{i,j}]\dot{f}({x}_{i,j})$ and ${\bar{x}}_{i,j}=({x}_{i-1,j}+{x}_{i+1,j})/2$ are the average values of the neighbouring intensity locations. $\dot{f}({x}_{i,j})=\frac{df(x)}{dx}={\sum }_{k}{c}_{k}{\dot{\varphi }}_{k}(x)={\dot{{\boldsymbol{\varphi }}}}^{T}(x){\bf{c}}$, being ${\dot{\varphi }}_{k}(x)=d\varphi (x)/dx$.

Upon the application of the fixed-point approach, the new locations of *j*^*th*^ profile, x_*j*_, can be obtained as follows

27 $${{\rm{x}}}_{j}^{t+1}=\frac{1}{3}({{\rm{\Omega }}x}_{j}^{t}-\frac{1}{\beta }{z}_{j})$$

Here, Ω is

28 $${\rm{\Omega }}=[\begin{array}{cccccc}1 & 2 & 0 & 0 & \ldots  & 0\\ 1 & 1 & 1 & 0 & \ldots  & 0\\ 0 & 1 & 1 & 1 & \ldots  & 0\\ \vdots  & \vdots  & \vdots  & \vdots  & \vdots  & \vdots \\ 0 & 0 & 0 & \ldots  & 2 & 1\end{array}]$$

and the term ${{\rm{\Omega }}x}_{j}^{t}$ represents a vector of the sum/average ${x}_{i-1,j}+{x}_{i,j}+{x}_{i+1,j}$ for each column profile x_*j*_. This equation is driven directly from (26).

As demonstrated in the Fig. 2B,C, this compensation strategy originates IN maps showing an almost constant horizontal invariant pattern of fluorescence that represents high levels of protein expression at the membrane and lower levels homogenously distributed at the cell cytoplasm. Confirming this observation, we verified that the maximum fluorescence intensity occurs at IN position 50 (Fig. 2C’), which corresponds to the plasma membrane shared between two contiguous cells (Fig. 2C”’). Further, when compared with the non-compensated IN profile, the compensated profile presents a smaller variance at each position and a higher sharpness of the peak (Fig. 2B’,B”,C’ and C”), demonstrating a significant improvement in image interpretation and quantitative analysis.

Regarding RD profiles, we verified that these maps can capture more efficiently protein distribution along the cell cytoplasm, which is not evaluated with IN profiling only (Fig. 2C”,E”). In fact, RD profiles were able to acquire the fluorescence signal into the full extent of a cell, revealing low levels of the marker inside the cell and its accumulation in cell periphery (Fig. 2E”,E”’).

These results indicate that the application of the alignment pipeline generates a rigorous profiling of image signals that can be statistically examined and virtually represented.

### Algorithm validation in IF images of heterogeneous cell populations stained for membrane, cytoplasmic and mitochondria markers

To experimentally validate our analytical pipeline, we used real *in situ* immunofluorescence images of cell cultures stained for E-cadherin, tubulin and mitochondria, which were selected as the prototypes of membrane, cytoplasmic and organelle-specific markers^15–18^.

**Figure 3:**
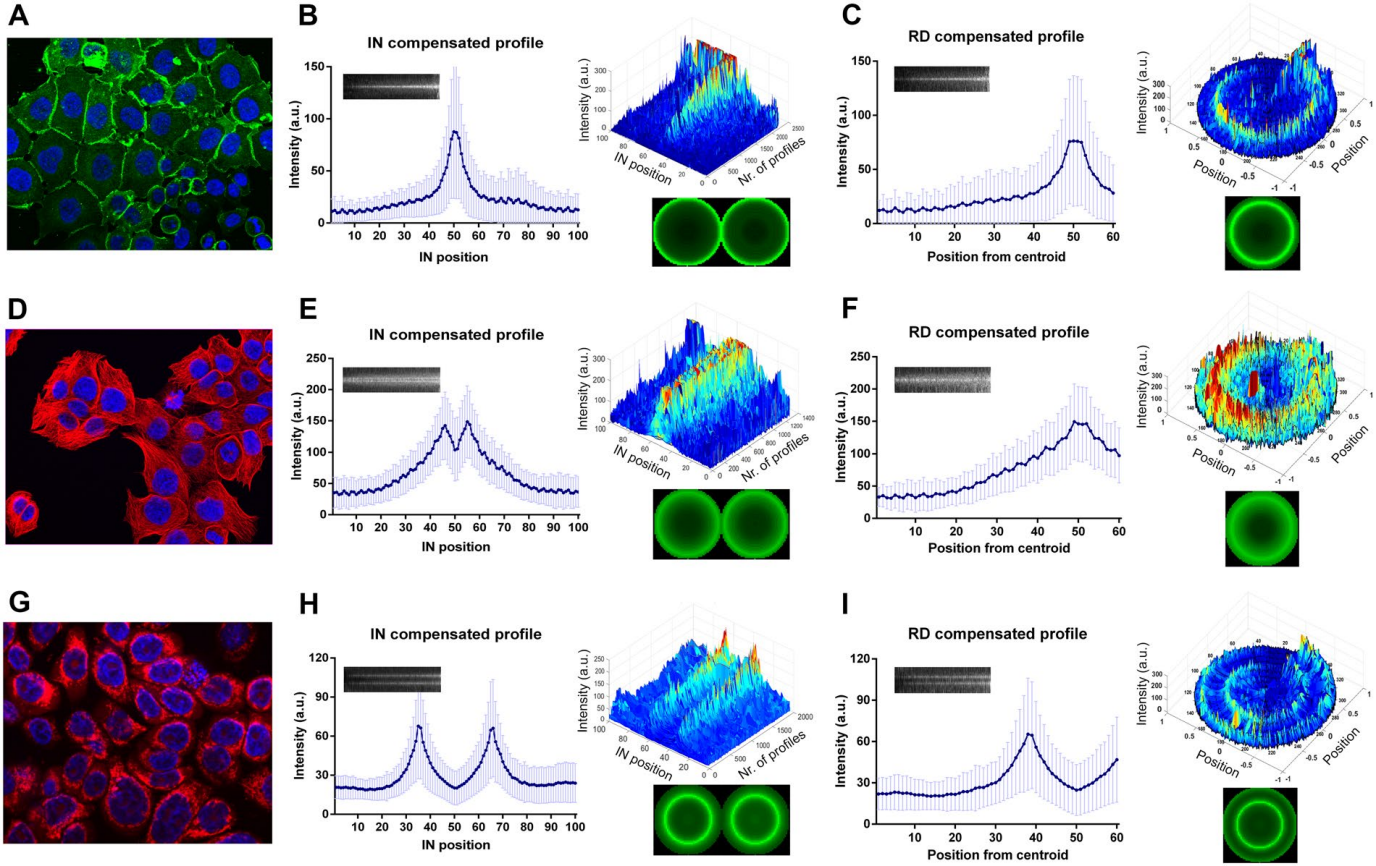
Method applicability in real immunofluorescence images of membrane, cytoplasmic and organelle-specific markers. (**A**) Immunofluorescence showing E-cadherin localization (green staining) in heterogeneous epithelial cells. Nuclei were counterstained with DAPI (blue). (**B**) E-cadherin IN profiles were extracted and geometrically compensated to evaluate protein distribution along contiguous cells. Compensated average intensity in each internuclear position ± SD and its corresponding IN compensated map are presented in the linear graph. 3D graph showing the overview of all extracted profiles upon compensation. Virtual cell pair construction based on IN compensated profiles. (**C**) Average of E-cadherin compensated RD profiles ± SD and its map are presented in the linear graph. Polar plot of all compensated RD profiles. 2D virtual cell illustrating E-cadherin distribution in the cell population. (**D**) Tubulin is stained in red and nuclei are marked in blue. (**E**) Average and map of tubulin IN compensated profiles. 3D graphical representation of IN compensated profiles and its virtual cell pair. (**F**) Average of tubulin RD profiles ± SD. Dynamic overview of all compensated RD profiles and its virtual illustration. (**G**) Mitochondria staining in red and nuclei marked in blue. (**H**) Average and map of mitochondria IN compensated profiles. 3D graph of IN compensated profiles and its virtual representation. (**I**) Mitochondria radial analysis including RD profiles mean ± SD. Overview of all compensated profiles and the respective virtual cell. a.u., arbitrary units.

In Fig. 3A, a cell culture showing E-cadherin membranous expression is presented. In this cell population, non-aligned profiles yield intense E-cadherin peaks randomly distributed in the cytoplasm, precluding true meaningful conclusions about protein status, namely its mapping and level of expression (Supplementary Fig. S1). Nonetheless, after geometric compensation, the analysis of IN maps revealed a strong intensity peak at the cell-cell junction (87,69 a.u. at IN position 50), in accordance with E-cadherin normal appearance and adhesive functional role (Fig. 3B). In addition, besides the membrane expression of the protein, compensated RD maps also detect the presence of lower E-cadherin levels diffusely distributed in the cytoplasm (Fig. 3C). This cytoplasmic protein fraction corresponds to the E-cadherin that is continuously being synthesised, recycled and degraded by mechanisms of protein trafficking that occur in several cytoplasmic organelles/structures, namely endoplasmic reticulum, golgi complex and endosomes^24–26^. Geometric compensation was also determinant for *in situ* image analysis of cytoplasmic and organelle specific proteins. For tubulin, a cytoskeleton component, it was possible to reveal a continuous increase in fluorescence from the position 20 till its maximum at the position 46, and a symmetric pattern between the positions 55 and 81 (Fig. 3D,E). Notably, at the position 50 that corresponds to the cell membrane, the protein level is reduced. This result is a precise overview of the IF image presented: a massive network is extended from the nucleus till a membrane-close region, where it polymerizes and accumulates. Indeed, microtubules are known to be present at the cytoplasma but not at the membrane^18^. The assessment of RD expression further confirmed these observations (Fig. 3F).

Regarding the mitochondria staining, the algorithm depicts a specific perinuclear expression pattern, either by IN or RD profiling (Fig. 3G–I). As showed in the dynamic IN and RD overviews, signals with maximum fluorescence intensities of 67,75 a.u. or 66,11 a.u. were restricted to a particular region of the cytoplasm that corresponds to positions 35 or 66 from the IN map (Fig. 3H,I). The remaining profile positions display much lower mean intensities (around 20 a.u.).

Overall, with this algorithm, we are able to achieve a precise and quantitative representation of the IF images of either membrane, cytoplasmic or organelle-specific markers, even in cases of highly heterogeneous cell cultures, such as those of cancer cells.

## Discussion

Despite all the advances in the bioimaging field, immunofluorescence remains the technique of choice to determine the level and the spatial and temporal changes of fluorescent-tagged molecules^2,27^. In the last years, an increased number of imaging methods have emerged, offering new possibilities to visualize and quantify fluorescent signals and allowing their dissemination in scientific research and clinical practice^3,27^. However, although major progress was achieved, the quantitative analysis of *in situ* cell fluorescence images still faces important limitations, being the morphologic variability within cell populations a major one^3,4^.

Cell morphologic variance/heterogeneity can result from a plethora of molecular events, such as changes in DNA sequence and status, distinct intercellular signalling, different cell cycle stages or altered cell behaviour^9,10^. Indeed, the size and the morphology of a cell can change abruptly upon acquisition of a DNA mutation or upon modulation of a single protein^28,29^.

In this work, we designed an analytical strategy to deal with cell shape and size variability that is based on a geometric compensation algorithm. With our approach, we were able to normalize cell size to a constant frame, and extract intensity profiles independently of cell morphological features. Ultimately, expression maps were generated reproducing an accurate level and a precise pattern of the target protein, in both synthetic and real cell cultures.

As a first step, we have computed two types of expression profiles, one focusing on cell-cell interactions (IN profiles) and the other directed to the distribution of a marker throughout the intracellular space of a single cell (RD profiles). The algorithm was shown to be successful in the extraction of both profile-types, nevertheless, their map compilation displayed scattered and undefined patterns of expression, which do not represent the typical profile of the cell population analysed. Therefore, an alignment model based on a geometric compensation algorithm was developed. By applying a classical image registration and alignment strategy, we set up a geometric compensation algorithm that detects common objects and reorient them in a way that corresponding data is paired^12,30^. This method enforced a controlled normalization of intensity maps and revealed a final set of compensated profiles from which we can estimate the ideal distribution of the protein in the internuclear or radial axes. At the end, a compensated map epitomizing the pattern observed in the original synthetic image was produced. In fact, the benefits of registration procedures have been demonstrated in diverse medical software by improving the visual or quantitative interpretation of the results from magnetic resonance image (MRI), ultrasound, positron emission tomography (PET), single photon emission computed tomography (SPECT) or magnetic resonance spectroscopy (MRS)^12,31^.

Subsequently, immunofluorescence of E-cadherin, tubulin and mitochondria was used to validate the applicability of our protocol in images of real cell cultures. Given that E-cadherin is the most important protein for the establishment and maintenance of cell-cell adhesion in epithelial tissues, it constitutes a classical example of a molecule strongly expressed at the plasma membrane^15,16^. In contrast, tubulin is the basic structure of microtubules, a major cytoskeletal component and, therefore, the prototype of an abundant cytoplasmic protein^32^. The performance of the algorithm was also tested in images of well-defined subcellular compartments, such as mitochondria, which is the organelle responsible for the cell energetic supply^19^.

For all the markers analysed, the method was successful. The results demonstrated that our strategy enables an accurate quantification and mapping of membrane, cytoplasmic and organelle-specific proteins. Upon length normalization and stacking of fluorescent profiles together in columns, we were able to recognize high protein expression in the middle position of the E-cadherin profile – in accordance with its normal membrane appearance. For tubulin, a clearly distinct phenotype could be noticed: increasing fluorescence levels along the cell cytoplasm till membrane-proximal regions, without any site-specific preference. Indeed, it is well-known that the cytoskeleton is extended all over the cell and it polymerizes close to the plasma membrane, sustaining cell shape and connecting all intracellular organelles by a dynamic network^18,32^. In contrast, in the case of mitochondria tracking, a sharp and intense peak is observed specifically in perinuclear positions, supporting its specialized and local metabolic function^19,20^. Overall, we demonstrate that this method is suitable to a large panel of molecules distributed in very distinct cellular compartments.

Although the protocol yields a homogenous view of the cell population analysed, cell lines presenting different protein distribution patterns (mixed populations) can be evaluated in a single-cell based approach. Given that each centroid (nucleus centre) composing the triangular network is numbered, its corresponding data can be easily identified and extracted. As so, instead of using mean values of the whole population, we are able to analyze each cell clone separately.

Most of the available methods for quantification of immunofluorescence images evaluate total intensity or number of pixels present in the fluorochrome channel, disregarding the expression profile of the target protein along the distinct subcellular compartments. Nevertheless, the recognition of abnormal patterns of expression can provide valuable information for research and for diagnostic purposes. For example, if a protein that is normally expressed at the membrane is being accumulated at the cytoplasm, as a result of trafficking deregulation mechanisms, the fluorescence levels can be the same although its pattern can be remarkably different and indicative of protein dysfunction^33^. In the last years, different bioimaging tools have been developed in an attempt to profile cell surface and intracellular markers along different cellular compartments^34,35^. Mosaliganti and colleagues engineered an automated method to first reconstruct membrane signals and then segment out cells from 3D membranes for quantification^36^. Still, the approach requires labelling of membrane boundaries and is only suitable for cell surface proteins^36^. Another protocol generates a coupled multidimensional representation of spatial distribution for nuclear and membrane-bound proteins in a process highly dependent on nuclear and membrane segmentation, as well as on the continuity of fluorescent signals along cell surface boundaries^37^. For the tracking of protein translocation between intracellular compartments, a system based on the average fluorescence changes over time was developed, using the variance instead of the raw fluorescence^38^. The tool allows the detection of a change in a compartment, even if the total amount of the dye remains unchanged but this strategy is limited to comparative analysis with an initial image^38^. In general, variation in background, signal discontinuity, non-uniformity in the width and strength of the signal, in addition to cellular morphological heterogeneity constitute major issues hampering successful analyses^34,35,37^. To overcome these limitations, cell segmentation procedures are usually required, increasing the complexity of the protocols and hindering their implementation and acceptance by biologists^34–36^.

To the best of our knowledge, this is the first description of a pipeline for imaging analysis, using a geometric alignment strategy, to investigate protein phenotypic signatures. Importantly, our computational method copes with cell-to-cell morphological differences, avoiding complex segmentation procedures or the need of continuous readouts of fluorescent signals. In summary, we propose a novel application of geometric compensation for a robust and automatic quantitative expression analysis (level and mapping) of either membrane or cytoplasmic proteins. This is a simple end-user method that can be widely explored in research and diagnostic labs to unravel protein regulation mechanisms or identify protein expression patterns associated with disease.

## Materials and Methods

### Cell culture

MKN28, MDA-MB-468 and CHO cells stably transfected with a vector encoding the wild type E-cadherin (as described previously)^33^ were cultured in RPMI, DMEM or α-MEM media (all from Gibco, Invitrogen), respectively, supplemented with 10% fetal bovine serum (HyClone, Perbio) and 1% penicillin/streptomycin (Gibco, Invitrogen). CHO Ecad cells were maintained under antibiotic selection with 5 μg/ml blasticidin (Gibco, Invitrogen). All cell lines were grown at 37 °C and 5% CO_2_ humidified air.

### Fluorescence staining

Cells were seeded on 6-well plates on top of glass coverslips and grown for 48 h, in order to reach 70% confluence. For E-cadherin immunofluorescence, fixation was performed in ice-cold methanol for 20 minutes, while for tubulin, cells were fixed in 4% formaldehyde for 30 minutes. Cells fixed in formaldehyde were treated with 50 mM NH_4_Cl for 10 minutes, washed with phosphate buffered saline (PBS), and permeablilized with 0.2% Triton X-100 in PBS for 10 minutes. Blocking was performed in 5% bovine serum albumin (BSA) in PBS for 30 minutes, at room temperature. Cells were, subsequently, incubated with E-cadherin mouse monoclonal (1:300 dilution, BD Biosciences) or anti-α-tubulin (1:1000, Sigma) antibodies for 1 h30. The Alexa Fluor 488 goat anti-mouse or the Alexa Fluor 594 goat anti-mouse (both diluted 1:500, Invitrogen) were applied for 1h in the dark, as secondary antibodies. MitoSOX Red (1.5 µM, Molecular Probes) specifically targeting mitochondria-derived superoxide anion was applied at 37 °C for 30 minutes in live cells. In this case, fixation was performed thereafter in 4% formaldehyde. Coverslips were mounted on slides using Vectashield mounting medium with DAPI (Vector Laboratories). Images were acquired on a Carl Zeiss Apotome Axiovert 200 M Fluorescence Microscope (Carl Zeiss, Jena, Germany) with an Axiocam HRm camera, and processed with the Zeiss Axion Vision 4.8 software.

### Synthetic images generation

Synthetic images mimicking heterogenous cell populations were generated in Matlab R2015b version. Geometric shapes, such as circles or ellipses, and free draw tools from the toolbox were used to produce reference patterns that simulate reorganization and distribution of cell-like objects. Intensity, contrast and hue were adjusted in each image, and Poisson noise was added in order to obtain synthetic images resembling fluorescence microscopy pictures.

### Nuclei segmentation and network generation

Denoising and nuclei segmentation were performed as previously described^21^. Images were first subjected to a pre-processing pipeline of contrast enhancement and adjustment of image intensities in order to diminish background and increase signal-to-noise ratios. Specifically, the Otsu method and the Moore-Neighbour tracing algorithm, modified by Jacob’s stopping criteria, were applied to each image for nucleus segmentation. In case of nuclei not properly segmented upon application of this protocol, nucleus manual fixation was performed using a computer-assisted mode. Nuclei geometric centre $({\boldsymbol{\upsilon }})$ was computed and its definition enabled the establishment of a segment connecting two neighbouring nuclei (***ε***) and, thus, the creation of an undirected graph, $G({\boldsymbol{\upsilon }},{\boldsymbol{\varepsilon }})$ by sequential association of other neighbours. A triangular network was designed using the Delaunay triangulation algorithm, which selects the mesh that maximizes the smaller angle of the triangles^39^. Highly obtuse triangles, with $\,{\phi }_{k} > {\mu }_{\phi }+3{\sigma }_{\phi }$ or ${\vartheta }_{k} > {\mu }_{\vartheta }+5{\sigma }_{\vartheta }$, were considered outliers and removed from the mesh.

### Data analysis

Data analysis and scientific graphing was performed through Graph Pad Prism software version 6.01 (Graph Pad Software, San Diego, CA) and Matlab R2015b.

### Computational power and process estimation

The analyses were performed in a regular computer with an Intel(R) Core(TM) i3 CPU M370@ 2.40 GHz, 6.00 GB RAM, 64-bit operating system and Windows 10 Home version 1709. The extraction of internuclear and radial profiles took less than 1 minute per image while the alignment and can take 5–20 minutes per image, depending on the number of cells that is present in each image. For large scale image analysis, intensity profiles should be first extracted for all images. Subsequently, a batch of mat-files containing non-aligned profile maps is submitted together for alignment under similar prior parameters.

## Electronic supplementary material

Supplementary Data
