# Supplementary material for: Geometric compensation applied to image analysis of cell populations with morphological variability: a new role for a classical concept
Source: Sci Rep. 2018 Jul 6;8:10266. doi: 10.1038/s41598-018-28570-z (PMC6035232; doi:10.1038/s41598-018-28570-z)
Supplement: Supplementary file 1 — Supplementary Data [file 41598_2018_28570_MOESM1_ESM.docx]

**TITLE**

**Geometric compensation applied to image analysis of cell populations with morphological variability: a new role for a classical concept**

Joana Figueiredo^1,2^, Isabel Rodrigues^3^, João Ribeiro^3^, Maria Sofia Fernandes^1,2,3^, Soraia Melo^1,2,4^, Bárbara Sousa^1,2^, Joana Paredes^1,2,4^, Raquel Seruca^1,2,4*^, João M. Sanches^3*^

^1^ Instituto de Investigação e Inovação em Saúde (i3S), Porto, Portugal;

^2^ Institute of Molecular Pathology and Immunology of the University of Porto (IPATIMUP), Porto, Portugal;

^3^ Institute for Systems and Robotics (ISR/IST), LARSyS, Bioengineering Dept, Instituto Superior Técnico, Universidade de Lisboa, Portugal;

^4^ Medical Faculty of the University of Porto, Porto, Portugal.

* Corresponding authors

**SUPPLEMENTAL DATA**

**
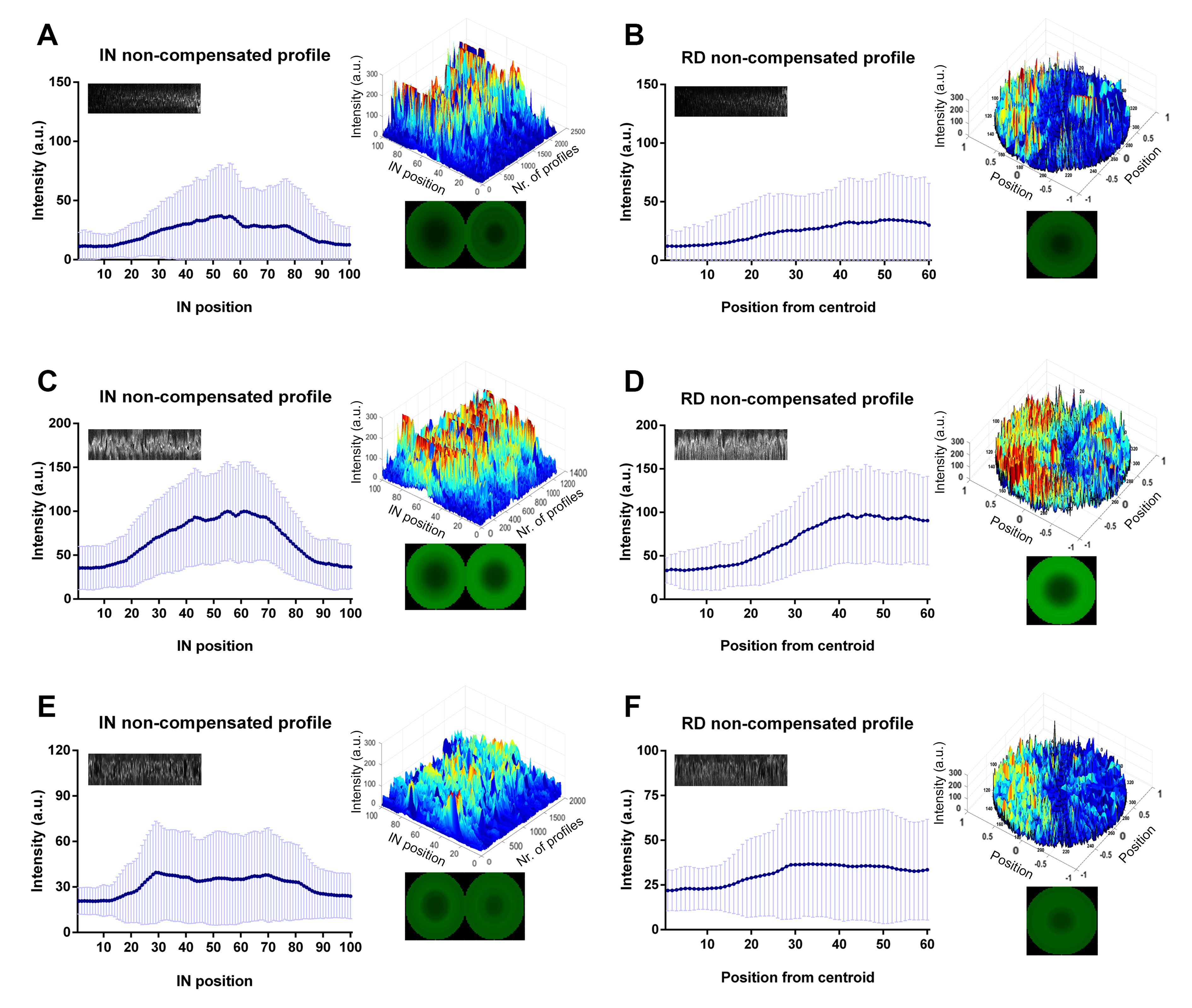
**

**Supplementary Figure S1. Non-compensated profiling of E-cadherin, tubulin and mitochodria stainings.** (A) Average intensity of E-cadherin non-compensated profiles in each internuclear position ± SD, and its corresponding IN map are presented in the linear graph. 3D graph showing the overview of all extracted profiles without compensation. Virtual cell pair construction based on IN non-compensated profiles. (B) Average of E-cadherin non-compensated RD profiles ± SD and its map are presented in the linear graph. Polar plot of non-compensated RD profiles and 2D virtual cell illustrating non-compensated E-cadherin distribution. (C) Average and map of tubulin IN non-compensated profiles. 3D graph of IN non-compensated profiles and its virtual cell pair. (D) Mean of tubulin non-compensated RD profiles ± SD. Overview of all compensated RD profiles and its virtual illustration. (E) Average and map of mitochondria non-compensated IN profiles. 3D plot of non-compensated IN profiles and its virtual representation. (F) Mitochondria RD profiles mean ± SD, overview of all non-compensated profiles and the respective virtual cell. a.u., arbitrary units.
